# Clinical Usefulness of Linked Color Imaging for Detection and Characterization of Undifferentiated-Type Early Gastric Cancer

**DOI:** 10.5152/tjg.2023.22684

**Published:** 2023-04-01

**Authors:** In Kyung Yoo, Hyuk Lee, Young Il Kim, Joo Young Cho, Wan Sik Lee

**Affiliations:** 1 Department of Gastroenterology, Cha Bundang Medical Center, Cha University College of Medicine, Seongnam, Republic of Korea; 2 Department of Medicine, Samsung Medical Center, Sungkyunkwan University School of Medicine, Seoul, Republic of Korea; 3 Center for Gastric Cancer, National Cancer Center, Goyang, Republic of Korea; 4 Department of Gastroenterology, CHA Gangnam Medical Center, Cha University College of Medicine, Seoul, Republic of Korea; 5 Division of Gastroenterology, Department of Internal Medicine, Chonnam National University Medical School , Hwasun, Republic of Korea

**Keywords:** Linked color imaging, early gastric cancer, undifferentiated

## Abstract

**Background:**

Linked color imaging is based on the bioluminescent imaging technique, which enhances differences in mucosal color allowing for contrast-based detection of lesions. There have been reports which have investigated the usefulness of linked color imaging for assessing color values in endoscopy for early gastric cancer cases. However, these primarily focused on differentiated early gastric cancer. This study aimed to assess the efficacy of linked color imaging in analyzing the color differences between cancerous and non-cancerous areas in undifferentiated-type early gastric cancer patients compared with conventional white light imaging.

**Methods:**

Forty-six patients were prospectively enrolled with undifferentiated-type early gastric cancer from 3 academic hospitals. All lesions were observed first by white light imaging followed by linked color imaging. An additional biopsy was taken from the surrounding mucosa to check for intestinal metaplasia, and test for *Helicobacter pylori* was performed. Color difference was measured in accordance with the International Commission on Illumination details.

**Results:**

The color difference value with linked color imaging was significantly higher, being more than twice that of white light imaging (26.82 ± 14.18 and 12.60 ± 6.42, *P* < .001), and this difference appeared to be similar in cases of accompanying *Helicobacter pylori* infection or intestinal metaplasia. In the subgroup analysis, color difference of poorly differentiated adenocarcinoma was notable in linked color imaging compared to white light imaging. Conversely, no statistically significant finding was present in signet ring cell carcinoma or mixed-type histology.

**Conclusion:**

Linked color imaging provides a significantly greater color difference between cancerous lesions and background non-cancerous mucosa in undifferentiated-type early gastric cancer. Moreover, linked color imaging may differentiate between pathologic subgroups of undifferentiated-type early gastric cancer possibly due to characteristic cellular growth pattern.

**Main Points**

- Linked color imaging (LCI) enhances the simultaneous expansion and reduction of color information, allowing for the reddish mucosa to appear redder and the whitish mucosa to appear whiter.
- Undifferentiated-type early gastric cancer (UD-EGC) tends to infiltrate the lamina propria without causing morphological changes to the surface; this limits the usefulness of narrow-band imaging for the identification of lesion boundaries when compared to differentiated cancers.
- This is the first study to examine the efficacy of LCI with regard to the color contrast between the undifferentiated-type gastric cancer and the surrounding mucosa.
- In this study, a comparison of the color difference between cancer and background mucosa surrounding the cancer in UD-EGC revealed that LCI demonstrated color difference levels to be twice than that of white light imaging.
- Linked color imaging may differentiate between pathologic subgroups of UD-EGC, possibly due to characteristic cellular growth pattern.

## INTRODUCTION

Although gastric cancer is the second most common cause of cancer-related deaths worldwide; a relatively high survival rate can be achieved with early detection.^1^ In particular, lesions with appropriate indications can be treated with minimally invasive procedures, such as endoscopic submucosal dissection (ESD).^2^ To reach this target, close monitoring of the lesion and identification of boundaries are crucial. In addition to conventional white light imaging (WLI), various image-enhanced endoscopic modalities have been introduced in the field of endoscopic therapy for close monitoring of lesions and better identification of lesion characteristics.^3,4^ Among these, narrow-band imaging (NBI) was reported to be effective in improving the visualization of microstructures and microvessels for the diagnosis of gastric lesions.^5^

The recently developed linked color imaging (LCI; Fujifilm Corp., Tokyo, Japan) is based on the bioluminescent imaging technique combined with digital image processing, which creates images by the emission of both white light and narrow-band short-wave light, processing images to indicate a well-separated red area.^6,7^ Linked color imaging enhances the simultaneous expansion and reduction of color information, allowing for the reddish mucosa to appear redder and the whitish mucosa to appear whiter. Given that the depth of color in LCI is more similar to that in WLI than in NBI, LCI is considered a more effective technique for separating mucosal colors. Recent studies have assessed the efficacy of LCI for diagnosing tumorous lesions in the upper gastrointestinal tract.^8-13^ Specifically, a small number of studies have reported LCI to be useful for assessing the color difference between cancerous and non-cancerous mucosa in early gastric cancer (EGC) cases. However, the results were mainly about differentiated cancers.^14-16^ While the standard treatment for undifferentiated-type EGC (UD-EGC) is surgery, mucosal cancer with lesions of ≤2 cm in diameter is included in the expanded indications for ESD.^17-19^ Therefore, accurate prediction of lesion size is very important. However, data regarding the effectiveness of image-enhanced endoscopy for demarcation of UD-EGC are scarce. Clinical data on the effectiveness of the most widely used technique, NBI, have reported that UD-EGC tends to infiltrate the lamina propria without causing morphological changes to microvessels and microscopic surfaces, thus making the identification of the lesion boundaries more difficult than that in differentiated cancer.^20^ Therefore, this study aimed to analyze the color difference between the malignant lesions and surrounding non-cancerous areas and compare LCI with conventional WLI in a cohort of UD-EGC patients.

## MATERIALS AND METHODS

### Patients

**Figure 1.**
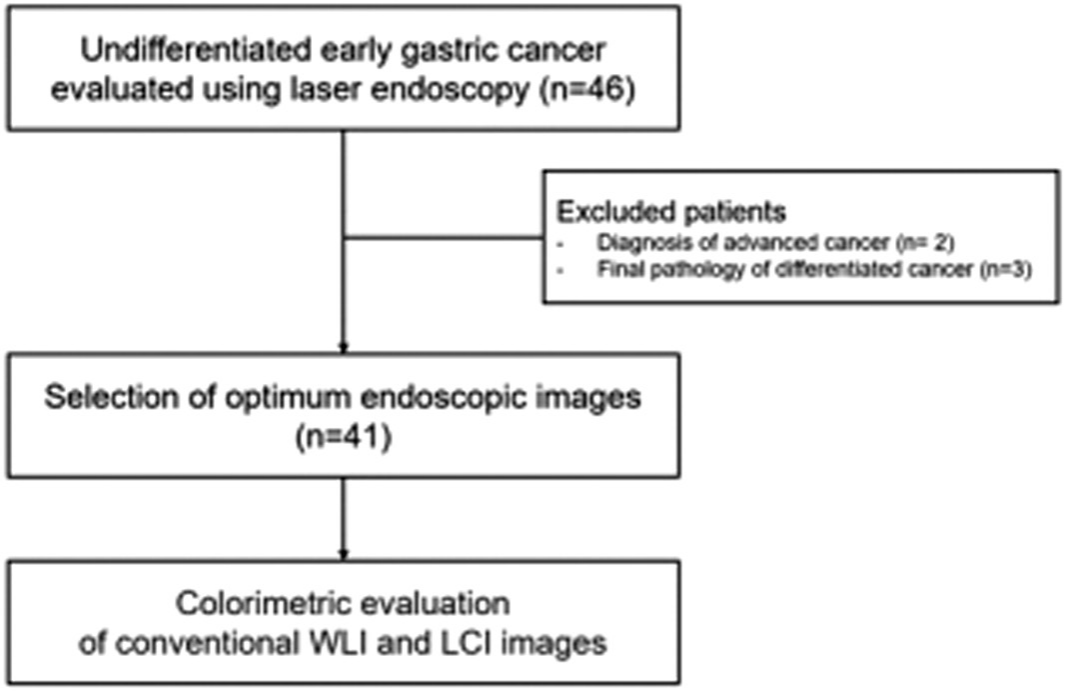
Flowchart of the study patients.

We enrolled 46 patients who were diagnosed with a UD-EGC based on biopsy from 3 hospitals (Chonnam National University Hwasun Hospital, Cha Bundang Medical Center, and National Cancer Center) between February and October 2021. The present study was conducted in accordance with the Declaration of Helsinki and was approved by the ethical committee of Chonnam National University Hwasun Hospital (registration number: CNUHH-2020-221). The process from patient enrollment to analyses is outlined in Figure 1. Informed consent was obtained from the patients after providing information on the purpose of the study and potential adverse events. White light imaging and LCI images were obtained using EG-760Z endoscopes and the ELUXEO endoscopes system (Fujifilm Co.). Structure enhancement function and color modes were set to H+2+4 and C1 in WLI mode and the B8 level and level 1 in LCI mode, respectively.

All lesions were observed first by WLI followed by LCI. All WLI and LCI images were not magnified and obtained from the same distance and angle. Moreover, close-view and distant-view images were obtained for all lesions. All assessments were performed by 3 endoscopists (LWS, YIK, and KYI) who have at least 5 years of experience in endoscopic examinations. An additional biopsy was performed for the mucosa surrounding the malignant lesions to check for intestinal metaplasia, while a rapid urease test was performed to diagnose *Helicobacter pylori* infection.

An independent expert endoscopist, who was not involved in this study and was blinded to the study details, with at least 5 years of experience, selected at least 5 WLI and LCI images for each lesion that were suitable for analysis.

### Analysis

Images were analyzed in accordance with the method used in a previous study by Fukuda et al.^16^ In all images, the color difference between the malignant lesions and background mucosa was assessed, and the color difference between WLI and LCI was compared. The color difference was analyzed in accordance with the International Commission on Illumination details, 1976 L*a*b* color space, using a commercially available image analysis software (Adobe Photoshop, Adobe Systems, San Jose, Calif, USA), as reported previously.^15,21^

**Figure 2.**
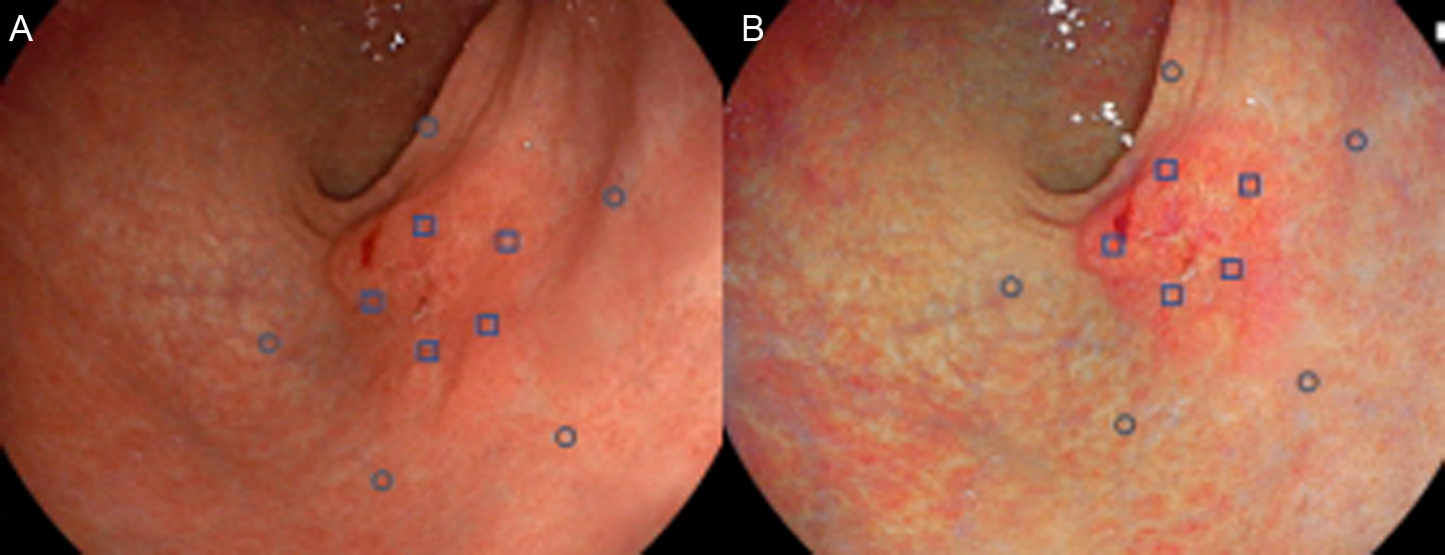
Analysis of color difference using the International Commission on Illumination 1976 color space. Five points were randomly selected from the cancerous lesion and nearby mucosa in white light imaging (A) and linked color imaging (B), respectively. The L*a*b* values of both cancerous lesion and surrounding mucosa were calculated based on the mean RGB values, and the color difference (Δ*E*) was calculated based on Δ*L*, Δ*a*, and Δ*b* using the formula ( 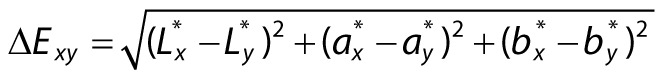 ). L* is defined as lightness, a* is the red–green component, and b* is the yellow–blue component. RGB, red, green, and blue.

In each of the 5 images from WLI and LCI, 5 points were randomly selected for the cancerous lesion and surrounding mucosa by a single independent endoscopist, responsible for image selection (Figure 2). The color difference was analyzed by another experienced endoscopist (LH). The mean red, green, and blue (RGB) values of the 5 points from the cancerous lesions and the background mucosa in the 5 images were calculated. The L*a*b* values of both malignant lesion and background mucosa were calculated based on the mean RGB values. The color difference (Δ*E*) was analyzed based on Δ*L*, Δ*a*, and Δ*b* values as described in previous studies.

### Pathological Diagnosis

All lesions were resected using ESD or surgical gastrectomy and pathologically assessed. The demarcation line between the cancerous lesion and normal mucosa was assessed in reference to the pathological examination of the resected specimen. Pathological diagnosis was made by a highly experienced pathologist based on the Japanese Classification of Gastric Carcinoma proposed by the Japanese Gastric Cancer Association.

### Statistical Analysis

The sample size of the study population was calculated considering the color difference between malignant lesion and non-malignant mucosa by WLI and LCI, as reported by Fukuda et al.^16^ The sample size required was calculated to be 38, based on the assumption of a power of 80% and an alpha risk of 0.05. Considering a dropout rate of 5%, the final sample size was determined to be 40 subjects.

The primary endpoint was color difference. Color difference is expressed as the mean (SD) value. For comparison of color difference between WLI and LCI, a 2-tailed paired *t*-test was used. For comparison of L*, a*, and b* values between the malignant lesion and surrounding mucosa, a 2-tailed unpaired* t*-test was used. *P* values of < .05 were considered significant. Data analyses were performed using Statistical Package for Social Sciences version 27.0 (IBM Corp.; Armonk, NY, USA).

## RESULTS

**Figure 3.**
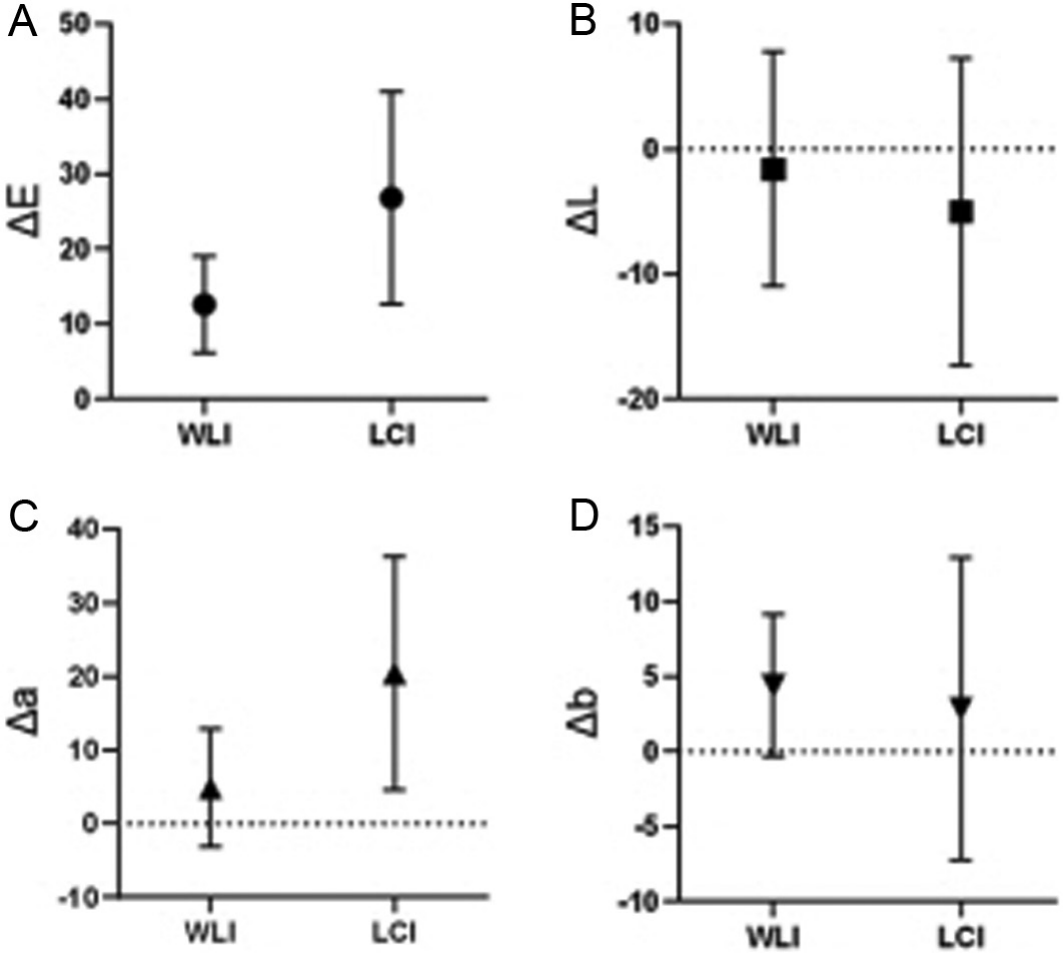
Color differences between cancerous lesions and the surrounding mucosa with white light imaging and linked color imaging.

**Figure 4.**
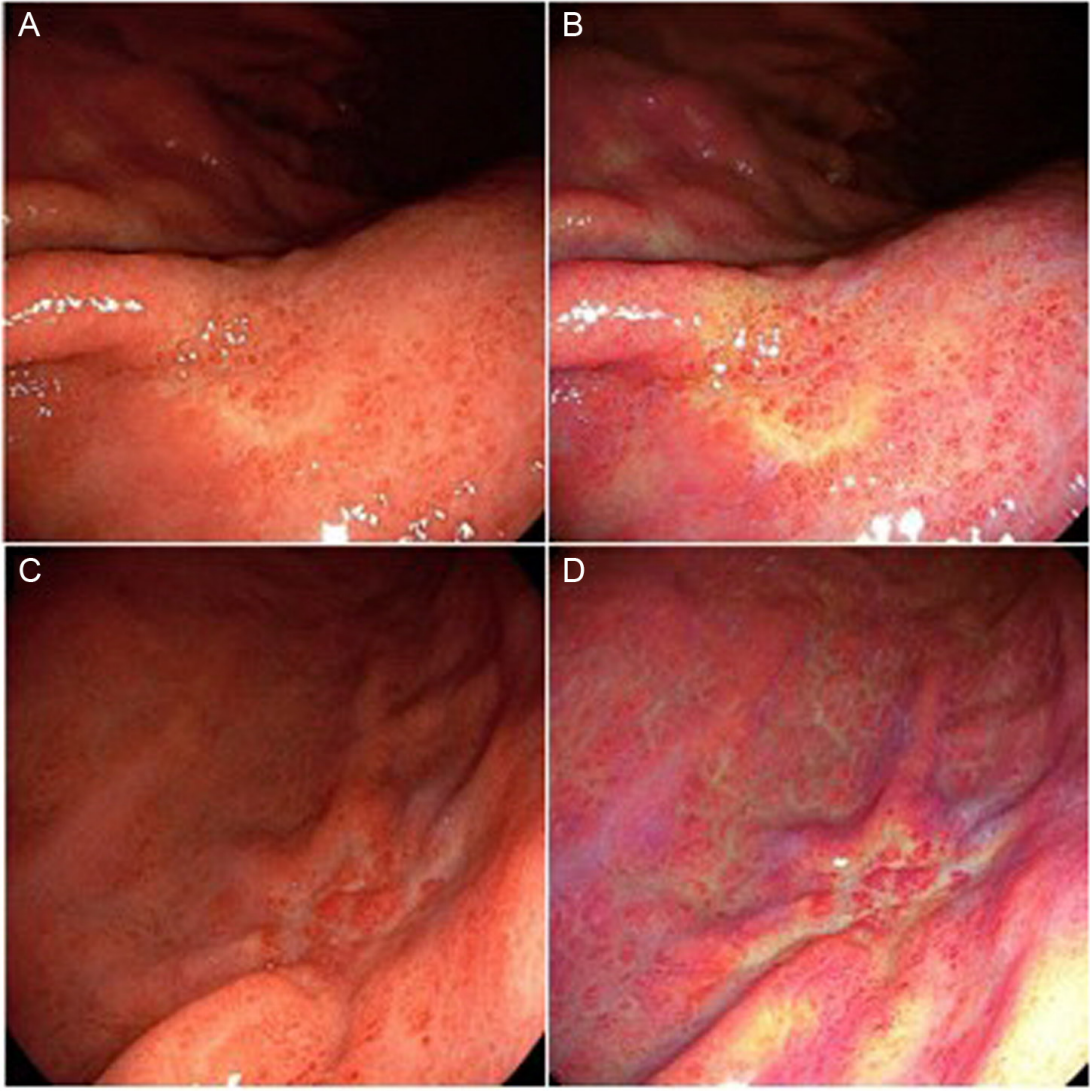
Representative endoscopic images of undifferentiated-type early gastric cancer. (A) WLI shows an ill-defined lesion at the posterior and greater curvature side in the gastric upper body. (B) On LCI, the lesion appeared as an orangish, depressed lesion with reddish-colored area in the central portion surrounded by purple-colored metaplastic mucosa. (C) A 2.5-cm-sized depressed lesion with reddish central area located in the posterior wall of the gastric mid body shown on WLI. (D) This lesion appeared orange-red in a background of diffuse reddish mucosa associated with* Helicobacter* gastritis using LCI. LCI, linked color imaging; WLI, white light imaging.

**Figure 5.**
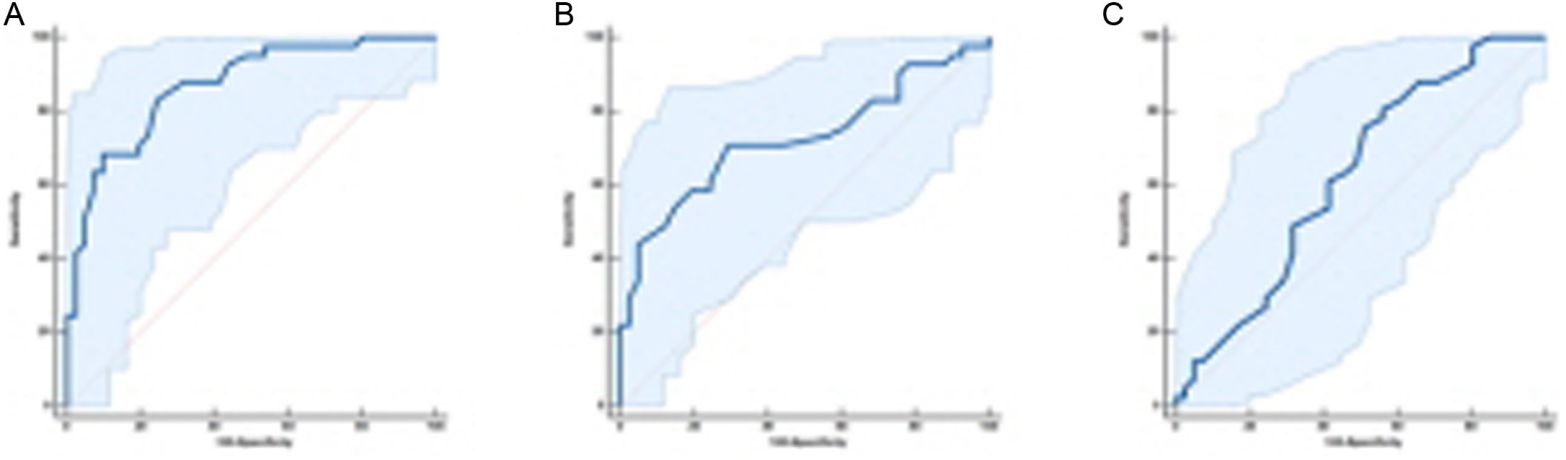
ROC curve demonstrating the predictive performance of color values (A: a*, B: b*, and C: L*) for the cancer in linked color images. ROC, receiver operating characteristic.

Forty-six lesions from 46 patients were enrolled before surgery or ESD. Five cases from all the enrolled cases were excluded predominantly because of diagnosis of advanced cancer after gastrectomy or pathological diagnosis of differentiated cancer. The images of 41 lesions from 41 patients corresponded to our inclusion criteria and were considered for color analysis. The patient and tumor characteristics in this study are shown in Table 1. The mean Δ*E* values with WLI and LCI were 12.60 ± 6.42 and 26.82 ± 14.18 (*P* < .001), respectively (Figure 3). The Δ*E* value with LCI was significantly higher than twice that of WLI. This high value was chiefly associated with an increase in the red–green component (a*) (20.43 ± 15.84 vs. 4.88 ± 7.98, *P*  <  .001). The malignant lesion showed a significantly higher color value than the surrounding mucosa in a* and b* in both WLI and LCI (Table 2). In subgroup analysis, the color difference significantly increased for elevated and depressed typed cancers, except flat type. A similar degree of color difference was observed in patients irrespective of *H. pylori* infections. Similarly, the Δ*E* value when using LCI was twice greater than that observed with WLI, regardless of the presence of the surrounding intestinal metaplasia around the tumor. However, the Δ*E* value by LCI did not appear to be higher in the cases of accompanying *H. pylori* infection or intestinal metaplasia. Representative images associated with the surrounding intestinal metaplasia and *H. pylori* gastritis are shown in Figure 4. We analyzed color differences in the subgroup according to final pathologic results. In poorly differentiated adenocarcinoma (PD), a marked difference in Δ*E* value in LCI compared to WLI (28.49 ± 14.93 vs. 12.63 ± 6.55, *P* < .001) was noted; however, in signet ring cell carcinoma (SRCC) or mixed type, the difference was not statistically significant. To elucidate the specific color value of cancer, the cutoff point was analyzed between the cancerous lesion and background mucosa in LCI using receiver operating characteristic curve analysis (Figure 5). The most suitable points of a*, b*, and L* were > 29, > 23, and ≤61, respectively. The sensitivity, specificity, and area under the curve were 82.9 %, 75.6%, and 0.87 for a*; 70.7 %, 70.7 %, and 0.72 for b*; and 75.6 %, 48.8 %, and 0.62 for L*, respectively.

## DISCUSSION

This study examined whether the diagnostic performance of LCI demonstrated a significant color difference to provide better clinical efficacy compared to that of conventional WLI for the detection of UD-EGC. Typically, EGC involves chronic inflammation in the mucosa surrounding the tumor, which may be missed by conventional WLI. To overcome this issue, alternative image-enhanced endoscopy may be effective.^6^ Narrow-band imaging is a common image-enhanced endoscopy technique which has been attempted to be used for the diagnosis and characterization of gastric cancer. Unlike the esophagus or colon, the gastric lumen has a large internal space which poses as a limitation as the space cannot be illuminated with sufficient light intensity.^22^ The clinical efficacy of LCI is highly anticipated to overcome this issue, as LCI offers a light intensity sufficient for examining the gastric lumen, enabling the detection of small gastric lesions in the distant view.^6^ Indeed, several studies have reported on the efficacy of LCI in observing EGC.[Table t3-tjg-34-4-356]

In 1 study, in a cohort of 52 patients with EGC, a greater color difference was observed between the cancer lesion and the mucosa affected by chronic gastritis surrounding it during measurement using LCI compared to that using WLI.^16^ Furthermore, similar outcomes were observed in the presence of intestinal metaplasia in the surrounding mucosa area. Moreover, another study demonstrated twice the color difference using LCI than WLI in EGC.^23^ Such improvements in color difference may have clinical implications for cancer detection rates. One randomized comparative study provided a prospective comparison of the detection rate of EGC between a group observed using both LCI and WLI and another group observed using WLI alone. A significant difference was reported between the groups, which demonstrated detection rates of 8.01% and 4.31%, in groups using both LCI and WLI and WLI alone, respectively, supporting the clinical efficacy of LCI.^24^ Moreover, a Japanese multicenter randomized trial compared the detection rates of LCI and WLI for the upper gastrointestinal tract and neoplastic lesions; it further demonstrated a significant difference in the detection rates at 8.0% and 4.8% in the LCI and WLI groups, respectively.^25^ As such, there is a high consensus among expert endoscopists for the use of LCI for differentiation between neoplasia and inflammation, while non-expert endoscopists regard LCI as an effective means to improve the yield of targeted biopsies.^8^

Most studies on gastric cancer are focused on differentiated EGC, making it difficult to apply such findings to UD-EGC. Although the current standard therapeutic approach for UD-EGC is surgery, endoscopic resection is another option included in the expanded indications for mucosal cancers with tumors <2 cm in size not accompanied by ulcers.^18,26^ Although endoscopic resection is indicated for differentiated types of intramucosal gastric cancer regardless of the tumor size, the tumor size criteria are important in UD-EGC.^27^ Delineating the gross and pathological sizes before and after endoscopic resection is an important factor, as incomplete delineation ultimately leads to noncurative resection.^28^ This warrants a more rigorous evaluation of tumor size before endoscopic resection in UD-EGC. Thus, there has been growing interest in the role of image-enhanced endoscopy in evaluating the size or characteristics of tumors in UD-EGC. Corkscrew patterns or the absence of microsurface patterns are characteristics of undifferentiated-type gastric cancer in magnifying endoscopy with NBI, but studies supporting these suggestions are limited.^29,30^ The few available studies have reported that magnifying endoscopy with NBI could improve the accurate diagnostic rate of UD-EGCs and demonstrate high accuracy in cancer demarcation.^31^ However, others have reported that the diagnostic accuracy of depressed- or flat-type UD-EGC is not significantly affected.^3,32^ Although few studies on alternative types of advanced imaging for the diagnosis of UD-EGC exist, the application of LCI for the above purpose has yet to be studied. Generally, it is presumed that the efficacy decreases in the presence of tumor infiltration below the normal mucosa in UD-EGC. It may cause the capillary vessel concentration to become unclear. Nevertheless, color enhancement of LCI may be expected to be beneficial in the detection and characterization of UD-EGC, although no clinical data on the color difference have yet been reported.

In this study, a comparison of the color difference between cancer and background mucosa surrounding the cancer in UD-EGC revealed that LCI demonstrated color difference levels to be twice than that of WLI. Such variance is often associated with differences in the red–green component and is present regardless of the presence of intestinal metaplasia in the surrounding mucosa or *H. pylori *infections. These results correspond with previous research findings on the role of LCI in differentiated cancer. As the inflammatory mucosa is visualized in purple color in LCI, in contrast to cancer tissue, which is usually orange-red in color, the color difference is stark, thus improving diagnostic accuracy. An interesting finding of this study was that a greater color difference due to LCI was evident in PD types, but the difference was not significant in SRCC types; thus indicating that efficacy may depend on histology. This is likely due to the different growth patterns of PD and SRCC types. A subepithelial spread of the tumor is characteristic of the SRCC type.^33,34^ Thus, there is greater margin involvement following endoscopic resection. For such pathological reasons, the prediction of horizontal margin by endoscopy is challenging for the SRCC type and seems to remain true in the context of LCI, as demonstrated in this study.

Although this is the first study to examine the efficacy of LCI with regard to the color contrast between the undifferentiated-type gastric cancer and surrounding mucosa, there are a few limitations. First, although the sample size of the study was predetermined to analyze the color difference, the primary efficacy variable, a larger sample size is likely needed for subgroup analysis. Namely, to examine differences based on the presence of metaplasia in the surrounding mucosa and histologic classification of the PD or SRCC types, the analysis must be performed on a larger target group. Second, although differences based on the presence of an *H. pylori* infection were analyzed, factors such as previous eradication history were not considered; thus, the possibility of a false-negative result cannot be excluded.

In summary, LCI presents a superior color difference between the cancer lesion and background non-cancerous mucosa in UD-EGC compared to that in conventional WLI, even in the presence of intestinal metaplasia or *H. pylori* infection in the surrounding mucosa. It is imperative that additional effects of examining UD-EGC using LCI should be validated through well-designed prospective comparison trial in the future.

## Figures and Tables

**Table 1.** Clinical Characteristics, Endoscopic, and Pathological Findings

Variables	Number (%)
Sex ratio (male/female)	19/22
Age, years, mean ± SD	63.9 ± 11.6
Tumor size, mm, mean ± SD	23.1 ± 10.1
Macroscopic type	
Elevated	7 (17.1)
Flat	5 (12.2)
Depressed	29 (70.7)
Final pathology	
SRCC	31 (75.6)
PD	5 (12.2)
Mixed	5 (12.2)
Depth	
Mucosa	34 (82.9)
Submucosa	7 (17.1)
*Helicobacter pylori *infection	
Negative	9 (22.0)
Positive	32 (78.0)
Surrounding intestinal metaplasia	
Negative	14 (34.1)
Positive	27 (65.9)

PD, poorly differentiated adenocarcinoma; SRCC, signet ring cell carcinoma.

**Table 2.** Color Values Between White Light Imaging and Linked Color Imaging in Cancerous Lesions and Non-cancerous Mucosa

Absolute Value	White Light Imaging	Linked Color Imaging	*P*
L*			
Malignant lesion	56.76 (9.56)	56.21 (8.30)	.7774
Surrounding mucosa	58.38 (11.51)	61.19 (11.11)	.2628
a*			
Malignant lesion	41.86 (8.81)	44.52 (12.85)	.1833
Surrounding mucosa	36.98 (7.99)	24.10 (11.96)	<.0001
b*			
Malignant lesion	37.33 (6.80)	23.21 (7.60)	<.0001
Surrounding mucosa	32.90 (6.72)	20.36 (6.98)	<.0001

L* is defined as lightness, a* is the red–green component, and b* is the yellow–blue component.

**Table 3. t3-tjg-34-4-356:** Color Differences Between Cancerous Lesions and the Non-cancerous Mucosa with White Light Imaging and Linked Color Imaging in Pre-specified Subgroups

Δ*E* in Subgroups	White Light Imaging	Linked Color Imaging	*P*
Morphology			
Elevated	10.73 (4.79)	24.03 (11.20)	.0025
Flat	12.91 (10.49)	30.59 (19.18)	.3840
Depressed	12.99 (6.09)	26.84 (14.25)	<.0001
*Helicobacter pylori* status			
Negative	12.49 (8.24)	30.30 (16.69)	.0011
Positive	12.63 (5.97)	25.84 (13.52)	<.0001
Intestinal metaplasia			
Negative	11.35 (4.10)	26.95 (14.22)	.0011
Positive	13.24 (7.33)	26.75 (14.42)	<.0001
Final pathology			
PD	12.63 (6.55)	28.49 (14.93)	<.0001
SRCC	9.76 (6.28)	16.30 (7.07)	.2109
Mixed	15.21 (5.63)	26.99 (11.34)	.0562

PD, poorly differentiated adenocarcinoma; SRCC, signet ring cell carcinoma.
